# Network reorganization of a cold-adapted β-glucosidase couples conformational flexibility to catalytic efficiency under low-temperature conditions

**DOI:** 10.3389/fmicb.2026.1821009

**Published:** 2026-07-27

**Authors:** Xian He, Mengting Liu, Xinwei Li, Liting Zheng, Derui Zhao, Peng Sang, Liquan Yang

**Affiliations:** 1 College of Agriculture and Biological Science, Dali University, Dali, Yunnan, China; 2 Key Laboratory of Bioinformatics and Computational Biology, Department of Education of Yunnan Province, Dali University, Dali, China; 3 Co-Innovation Center for Cangshan Mountain and Erhai Lake Integrated Protection and Green Development of Yunnan Province, Dali University, Dali, China; 4 College of Pharmacy, Dali University, Dali, Yunnan, China

**Keywords:** activity-stability trade-off, cold adaptation, conformational flexibility, enzymatic properties, enzyme dynamics, neural relational inference (NRI), β-glucosidase

## Abstract

**Introduction:**

Elucidating how cold-active enzymes maintain efficient catalysis under low-temperature conditions remains a significant question in enzymology. Although enhanced conformational flexibility has been frequently associated with cold adaptation, flexibility alone cannot fully explain how catalytic precision and reproducibility are maintained. This suggests that an additional layer of structural organization is required.

**Methods:**

In this study, a psychrophilic β-glucosidase (pBGL) from Pseudoalteromonas sp. BSw20308 and its mutant variant pBGL-S306P were examined through biochemical assays, molecular dynamics simulations, and dynamic interaction network analysis.

**Results:**

The results indicate that pBGL preserves high catalytic efficiency at low temperature despite pronounced thermal instability, maintaining a rigid and conserved catalytic core. Notably, increased flexibility is spatially redistributed toward peripheral and interfacial regions rather than being globally amplified. Network analysis reveals that cold adaptation is associated with a distributed interaction network that connects these flexible regions to the catalytic core, constraining motions into coordinated dynamics that support catalysis.

**Discussion:**

These findings suggest that cold adaptation in pBGL may involve not only enhanced flexibility but also network-mediated organization of conformational dynamics, providing a structural framework for understanding the activity–stability balance of cold-active enzymes.

## 1 Introduction

Low temperature imposes a fundamental constraint on biochemical reactions by reducing molecular motion and slowing reaction kinetics (Somero, 2004). As temperature decreases, both diffusion-limited encounters and transition-state crossing are expected to become progressively less favorable, posing a major challenge to enzymatic catalysis (Georlette et al., 2004). Nevertheless, enzymes from psychrophilic organisms can retain substantial catalytic activity under cold conditions, frequently accompanied by reduced thermal stability (Goldstein, 2007; Isaksen et al., 2014). This activity–stability trade-off has been observed in several cold-adapted enzymes from different enzyme families, suggesting that it may represent a recurring feature of cold adaptation rather than an isolated case (Pinney et al., 2021).

Understanding how enzymes maintain catalytic efficiency under low-temperature conditions, despite reduced molecular motion and slower conformational transitions and enzyme-substrate encounters, remains a central question in studies of cold adaptation (Cavicchioli, 2006; Nowak et al., 2024). Here, reduced thermal energy refers to the decrease in molecular thermal motion and slower conformational transitions that limit catalytic events under low-temperature conditions. Investigating how enzymes balance catalytic activity with reduced thermal stability is therefore important for elucidating the structural and dynamic bases of cold-active catalysis (Santiago et al., 2016).

A prevailing explanation for this challenge has centered on conformational flexibility (Feller and Gerday, 2003; Gerday, 2013). Numerous studies have shown that cold-active enzymes tend to exhibit increased structural plasticity and reduced thermal stability compared with their mesophilic or thermophilic counterparts (Pasi, 2009). This flexibility is commonly invoked to rationalize enhanced catalytic performance under low-temperature conditions, as increased structural mobility is thought to facilitate substrate binding, active-site rearrangement, and transition-state formation. However, while enhanced conformational flexibility is frequently associated with cold-active enzymes, it remains fundamentally a phenomenological description rather than a mechanistic explanation (Fields, 2001; Santiago et al., 2016). In particular, increased fluctuations alone do not account for how catalytically relevant motions are distinguished from conformational fluctuations ones, nor how catalytic efficiency is preserved without compromising specificity and control (Klinman, 2015). Thus, flexibility describes an outcome of cold adaptation, but leaves unresolved the deeper question of how enzymatic function is preserved.

If increased conformational flexibility alone was sufficient to explain cold adaptation, enhanced structural mobility would be expected to uniformly improve catalytic performance under low-temperature conditions (Åqvist et al., 2017). However, cold-active enzymes must still preserve substrate specificity and catalytic reliability despite increased fluctuations (Feller and Gerday, 1997). This apparent tension highlights a gap in flexibility-centered explanations of cold adaptation. Flexibility alone does not specify how catalytically relevant motions are distinguished from conformational fluctuations, nor how local structural changes may be coupled across distant regions of the protein (Schmid and Hugel, 2020). Because catalysis often depends on coordinated interactions among substrate-binding regions, catalytic residues, and distal structural elements, understanding how such dynamic organization is maintained under low-temperature conditions remains an important unresolved question (Fields, 2001).

Although increased flexibility has frequently been proposed as a feature of cold-active enzymes, its prevalence and functional significance remain subjects of ongoing discussion. Thus, the central unresolved question is not merely whether increased flexibility contributes to cold adaptation, but how such flexibility is spatially distributed and dynamically organized (Gerday, 2013; Collins and Feller, 2023). It remains unclear how conformational fluctuations are arranged across the protein in a way that supports catalytic function, rather than appearing as non-specific structural disorder, under low-temperature conditions (Jaenicke, 1990). Addressing this question requires moving beyond descriptions of local mobility toward a framework that captures enzyme-wide patterns of dynamic coupling and structural organization (Ghosh et al., 2024).

Cold adaptation may therefore involve not only increased conformational flexibility, but also the spatial organization of flexible regions within the protein structure. In this view, flexibility is not simply an end point of adaptation, but a property whose distribution may influence enzymatic function under low-temperature conditions (Somero, 2004; Ghosh et al., 2024). This perspective provides a useful framework for linking conformational flexibility with catalytic reliability without assuming that all correlated motions are necessarily functional. Productive catalysis depends on interactions among multiple spatially separated elements, including substrate-binding regions, catalytic residues, and distal structural motifs (Zhao et al., 2025). Network-level analysis can therefore help describe how local conformational fluctuations are coupled across the enzyme, although such coupling should be interpreted as descriptive rather than definitive evidence of catalytic causality. Through this dynamic organization, enzymes may preserve reliable function under low-temperature conditions, even when global stability is reduced (Agarwal, 2006).

To investigate this process, enzymatic assays were combined with molecular dynamics simulations and analyses of dynamic residue interaction networks using a pair of homologous β-glucosidases adapted to different thermal environments. In addition, a site-directed mutant, pBGL-S306P, was constructed to explore the effects of specific residue changes on network organization and conformational dynamics (Duan et al., 2025). This integrated approach enables the association of experimentally observed catalytic efficiency with patterns of conformational dynamics, providing a link between functional phenotypes and underlying structural flexibility. So far, the specific role of dynamic residue interaction networks in organizing flexibility for cold adaptation has not been directly examined.

## 2 Materials and methods

### 2.1 Gene cloning, protein expression, and purification

The gene encoding the cold-adapted β-glucosidase (pBGL) was identified from the genome of *Pseudoalteromonas* sp. BSw20308 (NCBI accession NO. AY646431.1). The coding sequence was amplified by PCR and cloned into the pET-28a(+) expression vector, introducing an N-terminal His tag to facilitate purification. The resulting construct was verified by DNA sequencing and transformed into *Escherichia coli* BL21 (DE3) cells for heterologous expression (Li et al., 2022).

Recombinant *E. coli* cells harboring the pET-28a-pBGL plasmid were cultured in LB medium supplemented with kanamycin (50 μg mL^−1^) at 37 °C until mid-log phase (OD_600_ ≈ 0.6–0.8). Protein expression was induced by adding IPTG to a final concentration of 0.2 mM, followed by incubation at 25 °C and 180 rpm overnight (Sadeghian-Rizi et al., 2019). Cells were harvested by centrifugation at 8,000 × g for 10 min at 4 °C and resuspended in PBS buffer (pH 8.0) containing 10 mM imidazole. Cell disruption was performed on ice using an ultrasonic cell disruptor (JY92-II, Ningbo Scientz Biotechnology Co., Ltd., China) at 150 W for 30 min, and insoluble material was removed by centrifugation at 12,000 × g for 30 min at 4 °C (Kruger, 2009; Li et al., 2022). The recombinant pBGL protein was purified from the clarified lysate using Ni^2+^-affinity chromatography under native conditions. The column was washed with buffer A containing 300 mM NaCl, 50 mM NaH_2_PO_4_, and 10 mM imidazole (pH 8.0), followed by further washing with buffer A containing 20 mM imidazole. Bound pBGL was eluted with buffer A containing 300 mM imidazole.

The recombinant pBGL protein was purified from the clarified lysate using Ni^2+^ affinity chromatography under native conditions. Bound proteins were eluted with an imidazole gradient, and fractions containing pBGL were identified by SDS-PAGE. The purity of the enzyme was confirmed by the presence of a single band at the expected molecular weight (~50 kDa; Supplementary Figure S1). Purified protein fractions were pooled and exchanged into storage buffer for subsequent biochemical characterization. Protein concentration was estimated using the Bradford assay with BSA as the standard (Zor and Selinger, 1996). The same expression, purification, and characterization protocols were applied to both the wild-type (pBGL-WT) and the S306P mutant (pBGL-S306P). Both variants exhibited identical optimal conditions for enzymatic activity, with an optimal temperature of 35 °C and an optimal pH of 7.0.

### 2.2 Enzymatic activity and kinetic characterization

Enzymatic activity of purified pBGL was assayed using cellobiose as the substrate. Each reaction mixture contained 10 μL of purified enzyme solution and 90 μL of reaction buffer containing 1% (w/v) cellobiose. Standard enzymatic activity assays were performed at pH 7.0 and 35 °C. Reactions were initiated by the addition of enzyme and incubated for 30 min. After incubation, reactions were terminated by heating at 90 °C for 10 min (Turner and Vulfson, 2000).

The amount of glucose released was quantified using a glucose oxidase–peroxidase (GOD–POD) colorimetric assay according to the manufacturer's instructions (Visvanathan et al., 2019). Following color development at 37 °C, absorbance was measured at 490 nm using a microplate reader. Enzymatic activity was calculated based on a glucose standard curve, and one unit of enzyme activity was defined as the amount of enzyme required to release 2.0 μmol of glucose per minute under the assay conditions. All measurements were performed in triplicate, and results are reported as mean ± standard deviation.

The effects of temperature and pH on enzymatic activity were evaluated by conducting assays over a range of temperatures and buffer conditions. Relative activity was expressed as a percentage of the maximum activity observed under the respective experimental series (Herlet et al., 2017). Thermal stability was assessed by pre-incubating the enzyme at the indicated temperatures for defined time intervals prior to activity measurement. Residual activity was determined under standard assay conditions and expressed relative to the untreated control. Apparent half-lives (t_1/2_) were estimated from exponential fits to activity decay curves.

Kinetic parameters were determined by measuring initial reaction rates at varying substrate concentrations. Reaction velocities were obtained from the linear range of product formation and fitted to the Michaelis–Menten equation using non-linear regression, and the fitted curves for pBGL at 25 and 35 °C are provided in Supplementary Figure S2 (Zainol and Ismail, 2019). The Michaelis constant (K_m_) and catalytic turnover number (k_cat_) were calculated based on estimated enzyme concentration from purified protein samples (Meyer-Almes and Auer, 2000). For comparison between temperature-adapted enzymes, kinetic measurements were performed using the same substrate where applicable and under conditions optimized for each enzyme (Meyer-Almes and Auer, 2000; Yang et al., 2020). Potential mutation sites were first screened using the HotSpot Wizard 3.0 web server (Sumbalova et al., 2018), which integrates structural, functional, and evolutionary information to identify residues suitable for protein engineering. Residue S306 was predicted as a promising target for improving enzyme stability and was therefore selected for site-directed mutagenesis.

### 2.3 Homology modeling and structural preparation

Because no experimentally determined structure is available for pBGL, a three-dimensional structural model was generated by homology modeling. The amino acid sequence of pBGL was used to search for structurally solved homologs in the Protein Data Bank (PDB; https://www.rcsb.org). A mesophilic β-glucosidase from *Exiguobacterium marinum* (PDB ID: 6WIU), which exhibits the highest sequence similarity to pBGL among all available experimentally solved GH1 structures, was selected as the template, considering both sequence similarity and conservation of the GH1 fold. In contrast, the β-glucosidase from *Exiguobacterium antarcticum* (PDB ID: 5DT5) was included only as a reference for visualizing structural features of cold-adapted enzymes. Sequence alignment between pBGL and the template (6WIU) was performed using Clustal X2 (Larkin et al., 2007), and multiple sequence alignment was visualized with ESPript (Gouet et al., 1999) for structural insight.

The three-dimensional structure of pBGL was predicted using AlphaFold2 (Jumper et al., 2021; Matinja et al., 2024). The resulting model exhibited a pTM score of 0.97, indicating a high confidence in the overall backbone conformation.

For comparative analyses, the experimentally determined structure of the mesophilic homolog mBGL (PDB ID: 6WIU) was used directly. Prior to molecular dynamics simulations, both pBGL and mBGL were prepared as protein-only monomeric structures. The prepared monomeric structures were subsequently used as starting coordinates for molecular dynamics simulations (Sang et al., 2016; Li et al., 2023).

### 2.4 Molecular dynamics simulations and structural characterization

Molecular dynamics (MD) simulations were performed to characterize the conformational dynamics of the cold-adapted enzyme pBGL and its mesophilic homolog mBGL. All simulations were carried out using GROMACS with the CHARMM36 force field (Abraham et al., 2015). The homology model of pBGL and the experimentally determined structure of mBGL were used as starting coordinates (Yang et al., 2014, 2020; Li et al., 2023).

Each protein structure was placed in a dodecahedral simulation box and solvated with the TIP3P water model, ensuring a minimum distance of 1.0 nm between any protein atom and the box boundary (Gupta et al., 2016; Sang et al., 2016). Counterions were added as needed to neutralize the system. Energy minimization was conducted using the steepest descent algorithm to remove unfavorable contacts, followed by equilibration under canonical (NVT) and isothermal-isobaric (NPT) ensembles with position restraints applied to protein heavy atoms (Yang et al., 2020). Temperature was maintained using a velocity-rescaling thermostat, and pressure was controlled at 1 bar using the Parrinello-Rahman barostat.

Production MD simulations were subsequently performed for 200 ns with a time step of 2 fs. Long-range electrostatic interactions were treated using the particle mesh Ewald (PME) method (Petersen, 1995), and van der Waals interactions were calculated using a cutoff of 1.0 nm with the Verlet scheme (Liu et al., 2023). All covalent bonds involving hydrogen atoms were constrained using the LINCS algorithm (Hess, 2008). Trajectory coordinates were recorded at regular intervals for subsequent analysis. Independent simulations were performed to ensure statistical robustness.

Structural and dynamical properties were analyzed using standard GROMACS utilities and complementary visualization tools (Abraham et al., 2015; Yang et al., 2020; Vieira et al., 2023). Backbone root mean square deviation (RMSD) relative to the starting structure was calculated to assess global conformational stability, while per-residue C_α_ root mean square fluctuation (RMSF) was computed to quantify local flexibility (Alkhatabi et al., 2025). Solvent-accessible surface area (SASA) was evaluated to characterize changes in protein compactness, and hydrogen-bonding interactions were analyzed to assess intramolecular packing and protein-solvent coupling (Abedi Karjiban et al., 2009; Sang et al., 2020). Together, these descriptors were used to characterize the spatial distribution of conformational flexibility and structural constraints in pBGL and mBGL.

The equilibrated MD trajectories obtained from these simulations served as the basis for subsequent dynamic interaction network inference and comparative analyses of temperature-dependent conformational organization.

### 2.5 Dynamic interaction network inference

To characterize how temperature affects residue-level dynamic coupling in pBGL and its mesophilic homolog mBGL, neural relational inference (NRI) was employed to reconstruct interaction networks from molecular dynamics trajectories (Zhu et al., 2022). NRI infers latent residue-residue dependencies based on time-resolved motion, enabling the description of dynamic coupling patterns beyond static proximity or contact persistence (Zhao et al., 2025).

For each system, including the wild-type enzyme pBGL-WT, the pBGL-S306P mutant, and the mesophilic enzyme mBGL, equilibrated molecular dynamics trajectories at 300 and 310 K were analyzed independently. From each trajectory, 5,000 conformational frames were uniformly sampled from the production phase. Each residue was represented as a node in the graph and described by the three-dimensional coordinates and velocity vectors of its C_α_ atom (Lovell et al., 2003). All input features were normalized to unit range prior to model training.

To construct training samples, trajectories were segmented into short temporal sequences of 50 consecutive frames, with a stride of 100 frames between segments. This sampling strategy balances temporal continuity with statistical independence while reducing redundancy. The NRI model was implemented using an encoder–decoder architecture, in which a variational graph encoder infers probabilistic interaction edges between residues and a decoder reconstructs residue trajectories based on the inferred network (Zhao et al., 2025). Model training was performed separately for each temperature condition using the Adam optimizer (Kingma and Ba, 2014) with an initial learning rate of 5 × 10^−4^ and a batch size of 1. Each model was trained for 500 epochs, with the learning rate reduced by a factor of two every 200 epochs to ensure convergence. The model checkpoint corresponding to the lowest validation loss was selected for network inference (Zhao et al., 2025).

Following training, probabilistic residue-residue interaction matrices were obtained by averaging inferred edge weights over all temporal segments. To reduce weak or noisy couplings, an interaction probability threshold was applied. For comparative analysis and visualization, residue-level interaction matrices were further coarse-grained into domain-level networks by averaging edge weights between residues belonging to the same structural region, as defined by secondary structure and functional interfaces (Zhao et al., 2025).

These temperature-resolved interaction networks provide a quantitative description of how dynamic coupling patterns change under different temperature conditions, allowing comparison of network connectivity and coupling strength among pBGL-WT, pBGL-S306P, and mBGL. For network visualization, inferred residue-residue and domain-level interaction networks were imported into Cytoscape (version 3.9.1) (Duan et al., 2025; Zhao et al., 2025). Nodes represent residues or structural domains, and edges represent inferred dynamic couplings, with edge thickness and color scaled according to the averaged interaction probability (Zhao et al., 2025). A force-directed layout was used to facilitate comparison of network topology and connectivity patterns across different temperature conditions. Network visualization was used solely for qualitative interpretation and illustration of temperature-dependent dynamic coupling patterns.

## 3 Results and discussion

### 3.1 Functional phenotype defines the cold-adaptation challenge

Enzymatic catalysis is influenced by temperature-dependent processes, including molecular diffusion, conformational transitions, and activation barrier crossing (Schwartz and Schramm, 2009). Under cold environmental conditions, these processes generally proceed more slowly, raising the question of how enzymes maintain sufficient catalytic activity. Establishing whether catalytic function is preserved under low-temperature conditions is therefore an important basis for analyzing the molecular features associated with cold adaptation (Somero, 1978; Schwartz and Schramm, 2009).

To address this question at the functional level, this study characterized the biochemical properties of a β-glucosidase (pBGL) derived from the psychrophilic bacterium *Pseudoalteromonas* sp. BSw20308. Recombinant pBGL was heterologously expressed and purified to homogeneity, yielding a single protein species with an apparent molecular weight consistent with the predicted size (~50 kDa; Supplementary Figure S1), enabling quantitative enzymatic characterization under defined conditions.

**Figure 1:**
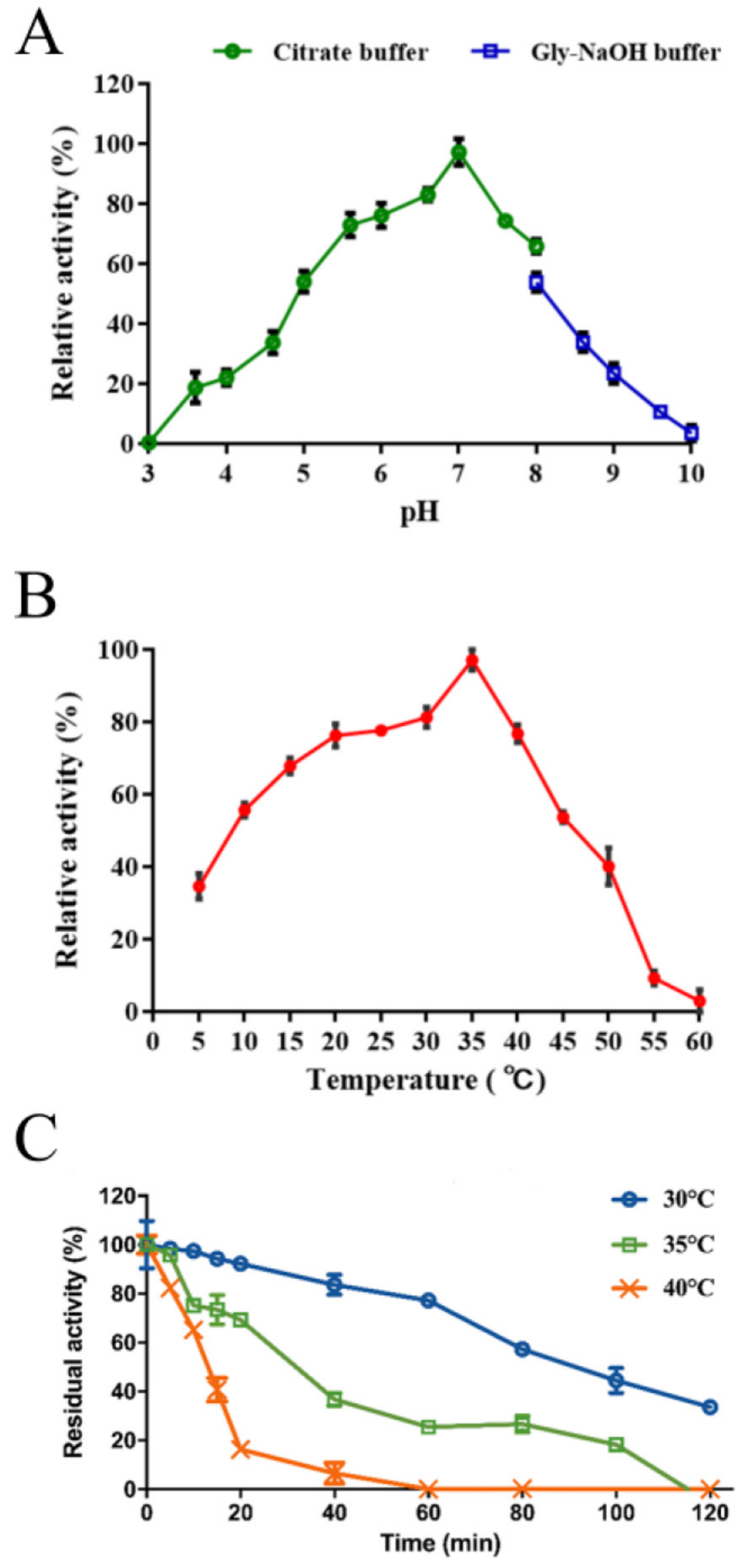
Functional characterization of the psychrophilic β-glucosidase pBGL reveals a pronounced activity-stability trade-off. **(A)** pH dependence of pBGL catalytic activity measured in citrate buffer (pH 5.0–8.0) and Gly-NaOH buffer (pH 8.0–10.0). Enzymatic activity at pH 7.0 was taken as 100%. **(B)** Temperature dependence of pBGL activity assayed over the indicated temperature range at pH 7.0. Activity measured at 35 °C was defined as 100%. **(C)** Thermal stability of pBGL assessed by residual activity following incubation at 30, 35, or 40 °C for the indicated times at pH 7.0. Residual activities were determined under standard assay conditions and normalized to the initial activity prior to incubation. All experiments were independently performed in triplicate using 1% (w/v) cellobiose as substrate. Data are presented as mean ± standard deviation.

Enzymatic activity assays showed that pBGL is catalytically competent across a broad temperature range, with maximal activity observed at near-neutral pH (Figure 1A). Its optimal assay temperature (~35 °C) is lower than that reported for the mesophilic homolog mBGL from *Exiguobacterium marinum* (~45 °C), and pBGL retained more than 80% of its maximal activity between 10 and 40 °C (Figure 1B). This broad activity window indicates that pBGL remains functionally robust under temperatures characteristic of cold environments.

Notably, this sustained catalytic performance was accompanied by pronounced thermal instability. Upon incubation at elevated temperatures, pBGL underwent rapid irreversible inactivation: more than 80% of enzymatic activity was lost within 20 min at 40 °C, and near-complete inactivation occurred within 120 min at 35 °C (Figure 1C). The corresponding half-lives at 35 and 40 °C were estimated to be 20.6 min and 10.6 min, respectively, underscoring the marked thermolability of pBGL relative to its mesophilic counterpart.

Kinetic analysis further demonstrated the favorable catalytic properties of pBGL. Using cellobiose as the substrate, pBGL exhibited a low Michaelis constant (K_m_ = 0.12 ± 0.02 mM), indicating strong substrate affinity. In addition, the turnover rate (K_cat_) measured at 35 °C was comparable to that of mBGL at its optimal temperature (Table 1). These results suggest that pBGL retains efficient catalytic performance at moderate temperatures and possesses favorable kinetic characteristics for cellobiose hydrolysis.

**Table 1:** Comparative biochemical and kinetic properties of the psychrophilic β-glucosidase pBGL and a mesophilic homolog.

Enzymes	Source of the enzyme, reference	Optimum (°C)	Assay temperature (°C)	Transition temperature	Optimum pH	K_m_, mM (Cellobiose)	_Kcat_ (s^−1^)	_Kcat_/K_m_ (s^−1^ mM^−1^)
pBGL	*Pseudoalteromonas* sp. BSw20308. (this work)	35 °C	35 °C	40 °C	7.0	0.12 ± 0.02	21.99 ± 2.05	180.58 ± 19.52
mBGL	*E. marinum* (de Sousa et al., 2020)	45 °C	45 °C	46 °C	6.5	34.54 ± 2.71	17.57 ± 1.57	0.51 ± 0.06

Values are reported as mean ± standard deviation (SD) from three independent experiments (n = 3).

The optimal temperature range refers to the temperature interval over which the enzyme retains 95%−100% of its maximal catalytic activity under standard assay conditions.

Transition temperature indicates the temperature at which a pronounced loss of enzymatic activity is observed during thermal incubation. For pBGL, the thermal inactivation half-life (t_1/2_) at 40 °C was determined to be 10.62 min.

Transition temperature indicates the temperature at which a pronounced loss of enzymatic activity is observed during thermal incubation. For pBGL, the thermal inactivation half-life (t_1/2_) at 40 °C was determined to be 10.62 min.

### 3.2 Distributed flexibility with a preserved catalytic core

**Figure 2:**
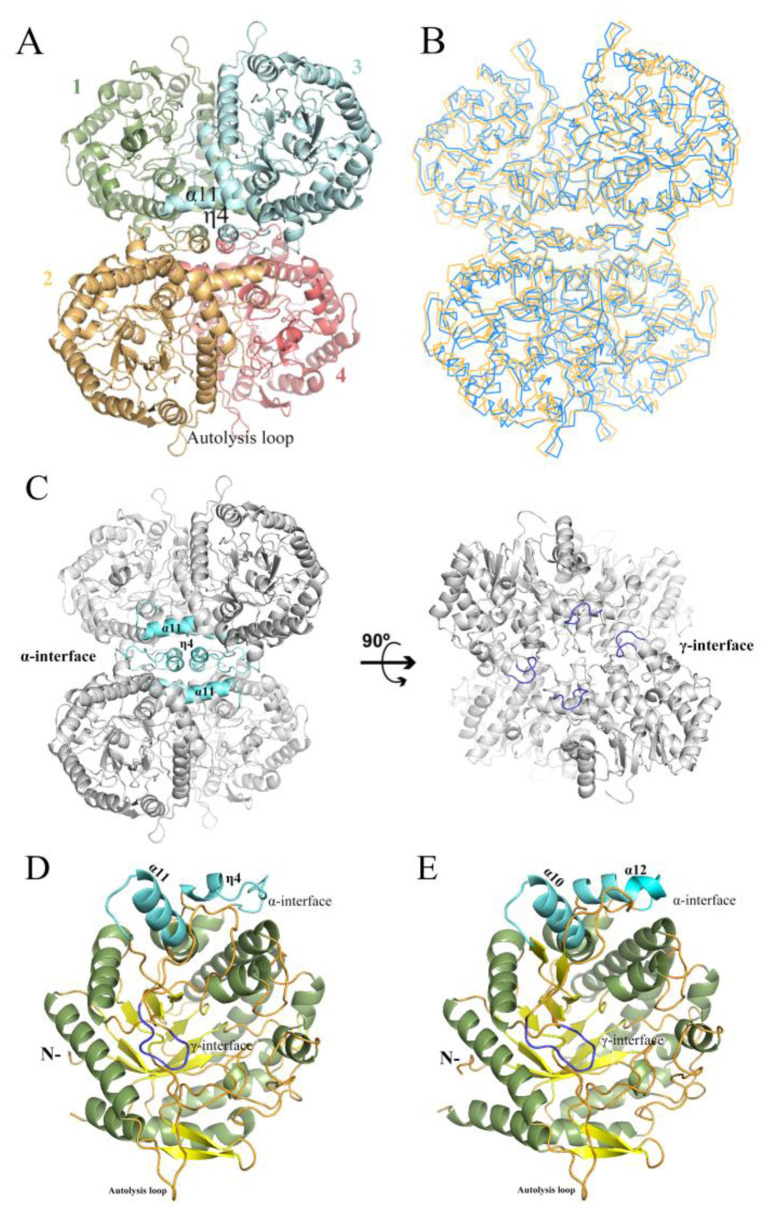
Structural organization of psychrophilic and mesophilic β-glucosidases. **(A)** Tetrameric assembly of pBGL from BSw20308 with the four monomers (1–4) shown in green, light blue, light orange, and red, respectively. The relative spatial arrangement highlights the inter-subunit interfaces formed on the catalytic face of the enzyme. **(B)** Backbone superposition of pBGL (blue) and its mesophilic homolog mBGL (light orange), illustrating the high degree of structural conservation despite differences in thermal adaptation. **(C)** Interfacial regions within the pBGL tetramer, highlighting the α-interface formed primarily by helices α11 and η4 **(left)** and the γ-interface on the catalytic face (**(right)**, rotated by 90°). These interfaces connect peripheral structural elements while remaining distal to the conserved catalytic core. **(D, E)** Monomeric structures of pBGL **(D)** and mBGL **(E)**, respectively. In both enzymes, the canonical (α/β) TIM-barrel scaffold and the catalytic core are preserved, whereas differences are primarily localized to interfacial and loop regions. In panels **(C–E)**, α-helices, β-strands, and loops are colored cyan, yellow, and light orange, respectively.

To examine how catalytic efficiency is maintained despite the pronounced thermal instability observed experimentally, the structural organization and intrinsic dynamic features of pBGL were analyzed in comparison with its mesophilic homolog mBGL. Because no experimental structure is available for pBGL, its three-dimensional structure was predicted using AlphaFold2. The resulting model retained the canonical (α/β) TIM-barrel scaffold typical of GH1 β-glucosidases (Figure 2A). Figure 2B shows the backbone superposition of pBGL and mBGL, highlighting their strong structural conservation. Figure 2C is a structural schematic of pBGL after a 90-degree rotation. Structural comparison with the experimentally determined mesophilic homolog mBGL (PDB ID: 6WIU) showed that pBGL shares substantial sequence identity with mBGL and preserves the conserved catalytic residues at the active site (Figures 2D, E). These observations suggest that the differences in catalytic behavior between the two enzymes are unlikely to arise from major alterations in the global fold or catalytic residue composition, but may instead be associated with differences in their dynamic organization.

**Figure 3:**
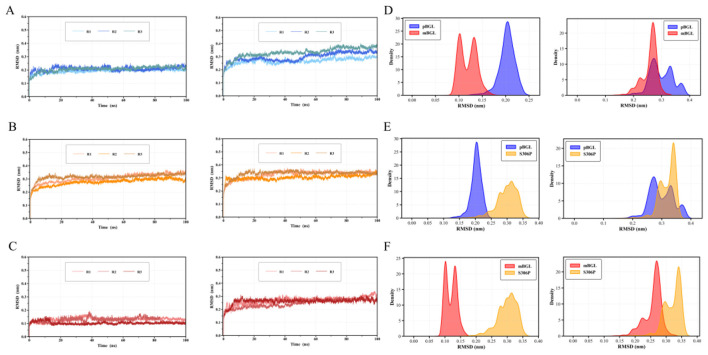
Temperature-dependent conformational stability of pBGL-WT, pBGL-S306P, and mBGL revealed by molecular dynamics simulations. **(A)** RMSD profiles of pBGL wild type (pBGL-WT) at 300 K **(left)** and 310 K **(right)**, shown in blue. **(B)** RMSD profiles of the pBGL-S306P mutant at 300 K **(left)** and 310 K **(right)**, shown in yellow. **(C)** RMSD profiles of the mesophilic enzyme mBGL at 300 K **(left)** and 310 K **(right)**, shown in red. **(D–F)** Probability density distributions of RMSD values for pBGL-WT, pBGL-S306P, and mBGL at 300 K **(left)** and 310 K **(right)**. The density plots indicate the proportion of conformational states occupying each RMSD range, thereby reflecting the conformational ensembles sampled by each enzyme under different temperature conditions. pBGL-WT is shown in blue, the pBGL-S306P mutant in yellow, and mBGL in red.

Despite this conserved catalytic framework, molecular dynamics simulations revealed differences in how structural fluctuations were distributed among pBGL-WT, the pBGL-S306P mutant, and mBGL. Analysis of backbone RMSD trajectories showed that all three systems reached relatively stable conformational ensembles at both 300 and 310 K (Figure 3). Compared with mBGL, pBGL-WT displayed larger deviations from its initial structure and broader RMSD distributions, indicating greater conformational variability under the simulated conditions. The pBGL-S306P mutant showed an intermediate dynamic profile, suggesting that this mutation partially altered the conformational behavior of pBGL. These results are consistent with the experimentally observed thermolability of pBGL and suggest that cold-adapted pBGL accommodates greater structural mobility than its mesophilic homolog.

**Figure 4:**
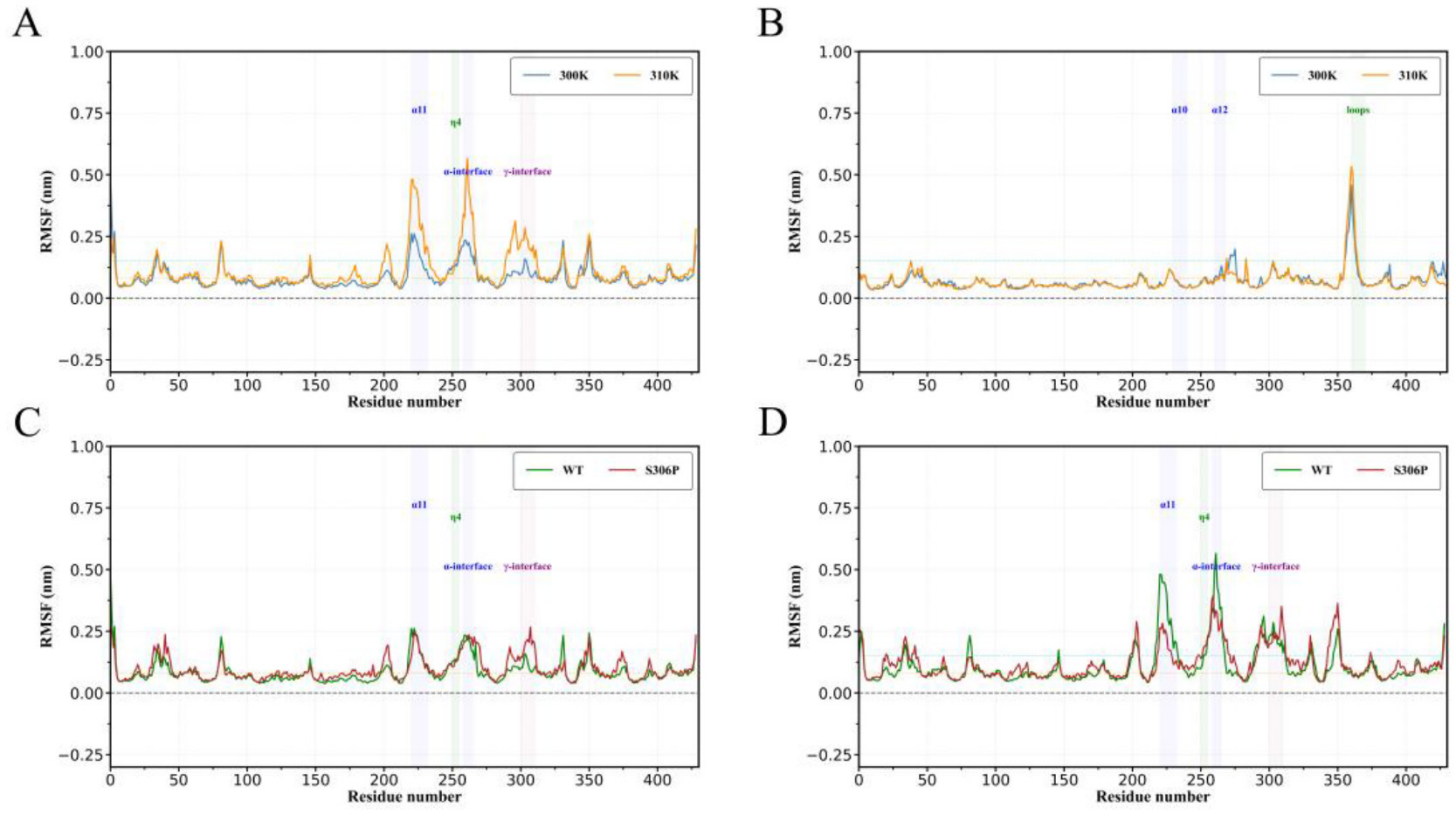
Spatial partitioning of conformational flexibility in pBGL-WT, pBGL-S306P, and mBGL. **(A)** Per-residue root-mean-square fluctuation (RMSF) profiles of Cα atoms for pBGL-WT at 300 K and 310 K, with 300 K shown in blue and 310 K shown in orange. **(B)** RMSF profiles of mBGL at 300 and 310 K, with 300 K shown in blue and 310 K shown in orange. **(C)** Comparison of RMSF profiles between pBGL-WT and the pBGL-S306P mutant at 300 K, with pBGL-WT shown in green and pBGL-S306P shown in red. **(D)** Comparison of RMSF profiles between pBGL-WT and the pBGL-S306P mutant at 310 K, with pBGL-WT shown in green and pBGL-S306P shown in red. RMSF values were calculated from equilibrated molecular dynamics trajectories and reflect the residue-level distribution of conformational flexibility under different temperature and mutation conditions.

Residue-level root-mean-square fluctuation (RMSF) analyses were performed for pBGL-WT, the pBGL-S306P mutant, and mBGL to examine how temperature affects local flexibility. In Figure 4A, RMSF values of pBGL-WT at 300 and 310 K are shown. With increasing temperature, fluctuations are enhanced, particularly at the α-interface formed by helices α11 and η4, the γ-interface lining the catalytic face of the TIM-barrel, and the N- and C-terminal segments. Figure 4B shows RMSF values of mBGL at 300 and 310 K, where most regions exhibit limited changes with temperature; only loop regions display relatively large fluctuations. Figure 4C compares RMSF values of pBGL-WT and pBGL-S306P at 300 K, showing similar flexibility profiles. Figure 4D shows the comparison at 310 K, where pBGL-WT exhibits overall higher fluctuations than pBGL-S306P, especially at the α11 helix and α-interface. These results indicate that conformational flexibility in pBGL is unevenly distributed and temperature-sensitive, while the catalytic core and most structural regions remain relatively stable across variants. The pBGL-S306P mutant exhibits slightly increased stability compared with the wild type.

The physical basis underlying this distributed flexibility becomes clearer upon examination of local structural descriptors. Relative to mBGL, pBGL exhibits a reduced number of intramolecular native contacts and intraprotein hydrogen bonds, together with a modestly increased solvent-accessible surface area (Table 2), indicative of weaker intramolecular packing and reduced structural compactness (Goldstein, 2007; Qing et al., 2022). Concomitantly, pBGL forms substantially more hydrogen bonds with surrounding solvent molecules across all simulated temperatures and throughout the equilibrated trajectories, reflecting enhanced protein–solvent coupling (Metpally and Reddy, 2009; Qing et al., 2022). This redistribution of stabilizing interactions from intramolecular contacts toward solvent-mediated interactions provides a structural basis for accommodating increased conformational mobility without compromising the integrity of the catalytic core.

**Table 2:** Global structural and interaction parameters of pBGL and mBGL derived from equilibrium MD simulations.

Protein	NNC^a^	SASA^b^ (Å^2^)	Rg^c^ (Å)	NHB^d^			RMSF^h^ (Å)
				Stat^e^	Dyna^f^	Protein-Solvent^g^	
pBGL	489,341 (3037)	19,747 (449)	21.1 (0.090)	310 (8.4)	1,953 (20.4)	10,106	0.127 (0.072)
mBGL	530,534 (2752)	18,756 (310)	21.3 (0.061)	358 (8.5)	2,087 (19.6)	9,132	0.069 (0.045)

^a^Number of native contacts. A native contact is considered to exist if the distance between the two atoms is less than 6 Å.

^b^Total solvent accessible surface.

^c^Radius of gyration.

^d^Number of hydrogen bonds. A hydrogen bond is considered to exist when the donor-hydrogen-acceptor is larger than 120° and the donoracceptor distance is smaller than 3.5 Å.

^e^Number of static hydrogen bonds for all frames.

^f^Number of dynamic hydrogen bonds in the trajectory.

^g^Number of hydrogen bonds in Protein-Solvent.

^h^Average RMSF value for all residues.

Taken together, the RMSD, RMSF, and SASA analyses suggest that cold adaptation in pBGL does not arise from a general loosening of the entire protein structure. Instead, pBGL adopts a dynamically partitioned architecture in which a rigid, conserved catalytic core is embedded within a more compliant peripheral framework. This organization allows conformational fluctuations to be spatially distributed toward interfaces and solvent-exposed regions, thereby expanding the accessible conformational ensemble while preserving catalytic geometry (Papaleo et al., 2016; Eberhart et al., 2025). While these analyses define where flexibility is accommodated within the enzyme, they do not describe whether fluctuations in different structural regions remain dynamically coupled. To explore this aspect, residue interaction networks inferred from molecular dynamics trajectories were analyzed as a complementary description of protein dynamics.

### 3.3 Network-level organization integrates distributed flexibility into coordinated catalytic dynamics

The molecular dynamics simulation analyses, including SASA, Rg, hydrogen-bonding patterns, dynamic state, and protein–solvent interaction energy analyses summarized in Table 2, suggest that cold adaptation in pBGL is characterized by pronounced, spatially distributed flexibility that is selectively accommodated at interfacial and solvent-exposed regions, while the catalytic core remains structurally restrained. However, enhanced flexibility alone does not fully explain how productive enzymatic dynamics are preserved. Distributed mobility, if left uncoordinated, would be expected to dissipate into stochastic fluctuations rather than support efficient catalysis. This therefore raises an important question: how are spatially dispersed conformational motions integrated into coherent, functionally productive dynamics (Bhabha et al., 2015)?

To address this question, residue-level and domain-level dynamic interaction networks for pBGL and its mesophilic homolog mBGL were constructed using Neural Relational Inference (NRI) (Kipf et al., 2018; Zhu et al., 2022; Zhao et al., 2025) applied to molecular dynamics trajectories across temperatures. This approach infers latent interaction strengths and directional couplings directly from time-resolved conformational fluctuations, providing a network-level representation of how local motions are dynamically coordinated (Zhao et al., 2025).

**Figure 5:**
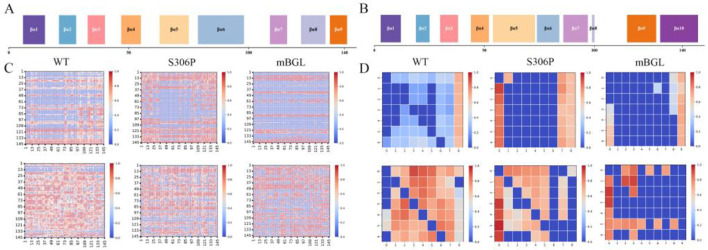
Network topology and inter-domain communication patterns of pBGL and mBGL inferred from NRI analysis. **(A, B)** Domain-partitioned strip representations of pBGL **(A)** and mBGL **(B)**. Residues were grouped into structural regions based on secondary-structure organization. Colored blocks represent distinct structural domains or regions. **(C)** Time-averaged inter-residue interaction matrices reconstructed from NRI models. The first row represents simulations at 300 K and the second row represents simulations at 310 K. The first column shows pBGL-WT, the second column shows the pBGL-S306P mutant, and the third column shows mBGL. Matrix elements represent the mean inferred interaction probabilities between residue pairs over the equilibrated trajectories. **(D)** Schematic summary heat maps of inter-domain interaction strengths derived from the NRI-inferred networks. The first row represents simulations at 300 K and the second row represents simulations at 310 K. The first column shows pBGL-WT, the second column shows the pBGL-S306P mutant, and the third column shows mBGL. Color intensity represents the relative magnitude of averaged coupling strength between domains.

Time-averaged interaction matrices highlight differences in residue-level network organization among pBGL-WT, the pBGL-S306P mutant, and mBGL across temperatures (Figures 5C, D). The corresponding domain-partitioned ribbon diagrams are shown in Figures 5A, B. At 300 K, pBGL-WT exhibits more broadly distributed interaction patterns than pBGL-S306P, which may be related to the proximity of 300 K to the favorable temperature range for pBGL-WT catalytic activity. When the temperature increases to 310 K, the interaction matrix of pBGL-WT shows a marked increase in overall coupling intensity. The pBGL-S306P mutant also shows enhanced interactions at 310 K, but the magnitude of this increase is less pronounced than that observed in pBGL-WT. In contrast, mBGL displays relatively stable interaction patterns between 300 and 310 K, with no obvious temperature-induced redistribution. These observations suggest that temperature modulates residue-level dynamic coupling more strongly in pBGL-WT than in pBGL-S306P or mBGL.

These contrasting patterns suggest that pBGL may adapt to high temperature by distributing interaction strength across multiple structural regions, thereby enabling extensive long-range communication, whereas mBGL may maintain function through localized rigidity and centralized coupling.

**Figure 6:**
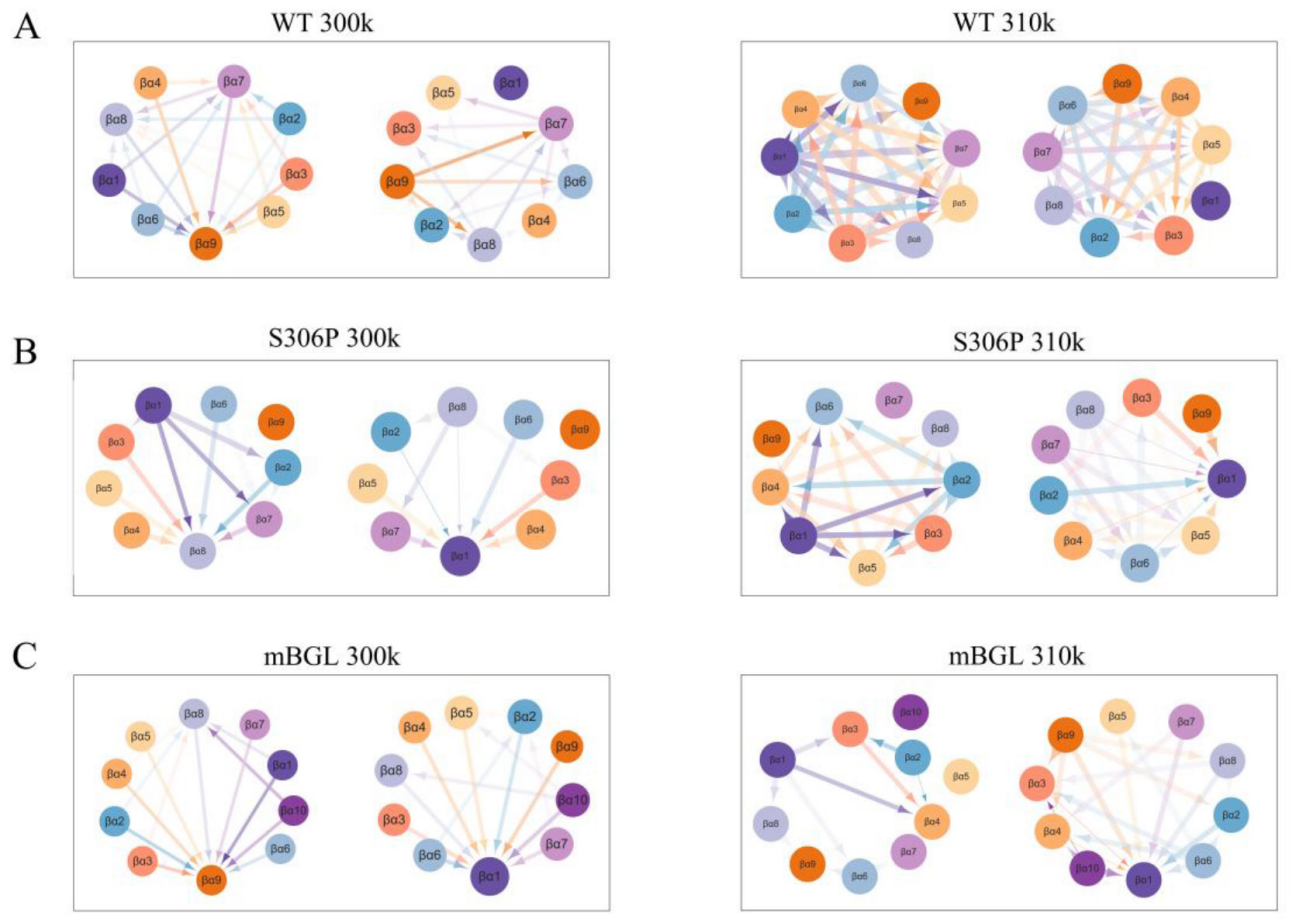
Temperature-dependent reorganization of domain-level interaction networks in pBGL-WT, pBGL-S306P, and mBGL inferred by NRI. **(A)** Domain-level interaction networks of pBGL-WT at 300 K **(left)** and 310 K **(right)**. **(B)** Domain-level interaction networks of the pBGL-S306P mutant at 300 K **(left)** and 310 K **(right)**. **(C)** Domain-level interaction networks of mBGL at 300 K **(left)** and 310 K **(right)**. Nodes represent structural domains, and directed edges represent inferred interactions between domains. Edge thickness reflects the relative interaction strength, while edge color indicates the direction of the inferred interaction. In each rectangular module, the left panel represents the first inferred interaction network and the right panel represents the second inferred interaction network; together, they constitute the complete interaction network of the corresponding structural domain. Networks are displayed using comparable layouts to facilitate direct comparison of interaction patterns among pBGL-WT, pBGL-S306P, and mBGL under different temperature conditions.

To further analyze how residue-level coupling patterns are organized at the domain level, interaction networks inferred from the NRI model were examined based on the custom-defined structural domains (Figure 6). At 300 K, pBGL-WT showed relatively strong domain-level connectivity involving βα1 and βα7, with additional connections to βα3, βα4, βα5, and βα6. This pattern suggests that several structural regions contribute to the dynamic coupling network rather than acting as isolated modules. At 310 K, the domain-level connectivity of pBGL-WT became more extensive, whereas the pBGL-S306P mutant showed a comparatively moderate increase in connectivity. In contrast, mBGL displayed relatively stable domain-level interaction patterns between 300 and 310 K. These results indicate that temperature-dependent changes in domain-level coupling are more pronounced in pBGL-WT than in pBGL-S306P or mBGL, although these network patterns should be interpreted as descriptive indicators of dynamic organization rather than direct evidence of functional communication.

As temperature increases from 300 to 310 K, pBGL-WT exhibits a progressive redistribution of domain-level connectivity. Additional peripheral nodes gain connections, and the network shows more diverse interactions, suggesting increased conformational flexibility. The pBGL-S306P mutant displays a similar trend but with a less pronounced increase in connectivity. In contrast, mBGL shows a relatively stable and sparsely connected network across both temperatures, with dominant nodes largely unchanged and peripheral regions contributing minimally to inter-domain coupling. These observations indicate that temperature-dependent changes in domain-level connectivity are more pronounced in pBGL-WT than in the pBGL-S306P mutant or mBGL.

Taken together, these results indicate that conformational flexibility in pBGL is not uniformly distributed but appears constrained by residue-level interactions. Distributed fluctuations observed in pBGL-WT are more pronounced than in the pBGL-S306P mutant or mBGL and are concentrated in peripheral and interfacial regions at 300 K. This organization may allow pBGL-WT to accommodate enhanced mobility while maintaining the integrity of the catalytic scaffold. At 310 K, interactions in pBGL-WT become more redistributed, with the pBGL-S306P mutant showing a moderate increase and mBGL remaining largely stable. These observations suggest that temperature-dependent changes in dynamic coupling are more prominent in pBGL-WT than in the mutant or mesophilic enzyme. They should be interpreted as descriptive measures of structural dynamics.

Thus, the key distinction between pBGL-WT, the pBGL-S306P mutant, and mBGL may not lie solely in the overall magnitude of flexibility, but also in how flexibility is distributed across the protein. In pBGL-WT, dynamic coupling patterns are more broadly distributed among structural regions, whereas mBGL exhibits comparatively localized interaction patterns. The pBGL-S306P mutant displays an intermediate organization between these two extremes. These observations suggest that differences in network organization may contribute to the distinct dynamic behaviors observed among the three enzymes.

## 4 Discussion

Cold adaptation remains an important challenge in enzymology. Low temperature generally slows molecular motion and reaction kinetics, yet cold-active enzymes can maintain substantial catalytic activity, often with reduced thermal stability (Santiago et al., 2016). Increased conformational flexibility has frequently been proposed as a contributing factor in this adaptation, and is often highlighted as a characteristic feature of certain cold-active enzymes (Siddiqui and Cavicchioli, 2006; Åqvist et al., 2017). Beyond the overall magnitude of flexibility, differences in the spatial distribution of conformational dynamics have also been observed among psychrophilic and mesophilic homologs (Karshikoff et al., 2015). In many proteins, structural fluctuations are distributed unevenly across different regions of the fold, reflecting distinct dynamic properties within the same protein structure (Henzler-Wildman and Kern, 2007). Therefore, examining how local mobility is distributed throughout the protein structure and comparing these patterns between cold-adapted and mesophilic enzymes may provide additional insight into their dynamic characteristics and adaptation-related structural features.

The results of this study indicate that cold adaptation in pBGL is associated with more than generalized structural flexibility. Despite thermal instability and elevated conformational fluctuations, the enzyme retains a relatively rigid catalytic core and a conserved active-site architecture. Molecular dynamics analyses further show that flexibility in pBGL is spatially partitioned, being more pronounced in interfacial and solvent-exposed regions rather than uniformly distributed across the fold. This pattern highlights the coexistence of region-specific flexibility and structural conservation within the same fold (Zhao et al., 2018). In addition, the observed dynamic features are accompanied by distinct residue interaction patterns, suggesting that conformational fluctuations in pBGL are organized differently from those in its mesophilic homolog (Collins and Feller, 2023).

More generally, network-level analyses provide a useful framework for describing how local fluctuations are arranged within the overall structural context of the protein. The dynamic interaction network reconstructed from the simulation trajectories provides a complementary structural context for interpreting these fluctuations (Zhu et al., 2022). In pBGL, cold adaptation appears to be associated with a reorganization of this network toward a more distributed and interconnected topology, with multiple communication routes linking distinct secondary-structure elements (Huang et al., 2023). Such a network architecture may facilitate long-range coordination among flexible regions while preserving coupling to the catalytic core. These observations suggest that flexibility is not merely increased in pBGL, but is reorganized within the interaction network, allowing local fluctuations to remain spatially coordinated rather than becoming broadly uncorrelated motions.

A key insight emerging from this analysis is that network organization may mediate the relationship between flexibility and structure (Zhu et al., 2022). In pBGL, increased connectivity among interfacial and peripheral regions may allow conformational variability to be integrated into more coordinated collective dynamics. Such distributed coordination may support catalytic function under low-temperature conditions, where thermal energy is limited and catalytically relevant motions must be efficiently utilized. At the same time, this network architecture may contribute to thermal vulnerability. As temperature increases, disruption of multiple weak interactions could promote network decoupling and accelerate the loss of structural integrity (Feller and Gerday, 2003). Consistent with this interpretation, the pBGL-S306P mutant exhibited enhanced thermal stability relative to the wild-type enzyme. Because proline generally restricts backbone mobility, this substitution may reduce excessive local flexibility and improve network coherence, thereby mitigating thermally induced network fragmentation (Matthews et al., 1987).

These observations suggest that modulating the balance between flexibility and connectivity through targeted mutations can partially shift the activity–stability trade-off characteristic of cold-adapted enzymes (Papaleo et al., 2011). Nevertheless, the organized yet flexible interaction network observed in pBGL may provide a structural basis for maintaining catalytic efficiency at low temperatures. By contrast, the mesophilic homolog mBGL appears to possess a more centralized and rigid interaction network dominated by strong couplings within the catalytic core. Such an organization may favor thermal robustness and structural persistence at elevated temperatures, but may also reduce the redistribution of flexibility that supports efficient catalysis under cold conditions (Qiao et al., 2017).

Taken together, the analyses presented in Sections 3.2 and 3.3 support a structural framework in which cold adaptation is associated not simply with increased flexibility, but with the spatial organization of conformational dynamics (Papaleo et al., 2013). In pBGL, flexibility is preferentially redistributed toward peripheral and interfacial regions, while the catalytic core remains relatively stable. Network analyses further suggest that these distributed motions are integrated through coordinated residue interactions, potentially enabling efficient communication between flexible regions and the active site (Doshi et al., 2016; Stetz and Verkhivker, 2017). Although the correlations derived from molecular dynamics simulations are descriptive and do not establish direct causal relationships, they provide a plausible structural explanation for how catalytic function can be maintained under low-temperature conditions (Somero, 1978; Knight et al., 2023). Within this framework, the activity–stability trade-off characteristic of cold-adapted enzymes may arise from the same dynamic properties that enhance functional coordination at low temperatures while simultaneously reducing resilience to thermal perturbation (Sydykova and Wilke, 2017). These findings provide a basis for future experimental studies aimed at testing the role of network organization in regulating enzyme adaptation and stability.

Beyond cold adaptation, these observations may provide a useful perspective for understanding enzyme dynamics and evolution. By emphasizing the spatial organization of conformational fluctuations rather than their overall magnitude, the network-based analysis suggests that differences in interaction topology may contribute to functional diversification among related proteins (Atilgan et al., 2004). More broadly, these findings support the view that enzyme function emerges from coordinated motions distributed across multiple regions of the protein rather than solely from individual structural elements (Agarwal, 2006; Ghosh et al., 2024).

In summary, this study suggests that cold adaptation in β-glucosidase is associated with network-level differences in the organization of conformational flexibility. The results indicate that distributed motions and dynamic interactions may contribute to maintaining catalytic function under low-temperature conditions (Jangid et al., 2025). Although these observations are consistent with the activity-stability trade-off commonly observed in cold-active enzymes, the causal relationship between network organization and enzymatic performance remains to be established (Feller and Gerday, 2003; Siddiqui and Cavicchioli, 2006). By integrating analyses of structural flexibility and dynamic interaction networks, this work provides additional insight into the molecular features associated with enzymatic adaptation to cold environments.

## Data Availability

The original contributions presented in the study are included in the article/Supplementary material, further inquiries can be directed to the corresponding author.
