# Supplementary material for: Network reorganization of a cold-adapted β-glucosidase couples conformational flexibility to catalytic efficiency under low-temperature conditions
Source: Front Microbiol. 2026 Jul 27;17:1821009. doi: 10.3389/fmicb.2026.1821009 (PMC13454050; doi:10.3389/fmicb.2026.1821009)
Supplement: Supplementary file 1 [file Supplementary_file_1.docx]

**Figure S1. SDS–PAGE analysis of recombinant pBGL expressed in *Escherichia coli*.** Lane 1, molecular weight markers (kDa) from Liji Biotechnology, with approximate molecular masses indicated on the left; Lane 2, purified recombinant pBGL; Lane 3, crude enzyme extract (supernatant after cell lysis). Proteins were resolved by 10% SDS-PAGE and visualized by Coomassie Brilliant Blue staining.

**Figure S2. Effect of temperature and mutation on the kinetic properties and stability of pBGL.**

To evaluate the effect of temperature on the kinetic behavior of pBGL, enzyme activities toward cellobiose were measured at different assay temperatures and fitted using the Michaelis–Menten model in GraphPad Prism. (A) Michaelis–Menten plot of pBGL at 25 °C, yielding a V_max_ of 1.410 μmol min^⁻¹^ and a K_m_ of 1.753 mg mL^⁻¹^. (B) Michaelis–Menten plot of pBGL at 35 °C. Data points represent experimental measurements, and solid curves represent nonlinear regression fits. (C) Thermal stability of wild-type pBGL (WT) and the S306P mutant at 35 °C. Enzyme samples were incubated at 35 °C for the indicated times, and residual activities were determined under standard assay conditions. Residual activity is expressed as a percentage of the initial activity at time zero. The specific activities of WT and S306P mutant under the standard assay conditions were 57.8 ± 1.3 and 41.7 ± 3.0 U/mg, respectively.

**Figure S3. Multiple sequence alignment of pBGL and mBGL.** Amino acid sequences of pBGL and the mesophilic homolog mBGL from Exiguobacterium marinum were aligned using Clustal X2. Secondary-structure elements are shown above the alignment.

**Figure S4. Structural features of pBGL highlighting catalytic residues and interfacial regions.** Cartoon representation of pBGL showing the conserved catalytic residues Glu170 and Glu352 rendered as stick models. The α-interface is highlighted in cyan, and the γ-interface is shown in blue, while the remaining regions of the protein are depicted in grey.

**Table S1.** Sequence information and amino acid composition of pBGL and mBGL

| **Enzyme Source** | **pBGL** | **mBGL** |
| --- | --- | --- |
| PDB ID | Homology model^a^ | 6WIU |
| Number of amino acids | 442 | 445 |
| Ala (A) number (ratio) | 35(7.9%) | 34(7.6%) |
| Arg (R) number (ratio) | 16(3.6%) | 21(4.7%) |
| Asn (N) number (ratio) | 21(4.8%) | 24(5.4%) |
| Asp (D) number (ratio) | 31(7.0%) | 40(9.0%) |
| Cys (C) number (ratio) | 5(1.1%) | 5(1.1%) |
| Gln (Q) number (ratio) | 13(2.9%) | 6(1.3%) |
| Glu (E) number (ratio) | 26(5.9%) | 33(7.4%) |
| Gly (G) number (ratio) | 31(7.0%) | 32(7.2%) |
| His (H) number (ratio) | 18(4.1%) | 20(4.5%) |
| Ile (I) number (ratio) | 27(6.1%) | 27(6.1%) |
| Leu (L) number (ratio) | 40(9.0%) | 27(6.1%) |
| Lys (K) number (ratio) | 27(6.1%) | 23(5.2%) |
| Met (M) number (ratio) | 10(2.3%) | 15(3.4%) |
| Phe (F) number (ratio) | 17(3.8%) | 27(6.1%) |
| Pro (P) number (ratio) | 20(4.5%) | 14(3.1%) |
| Ser (S) number (ratio) | 22(5.0%) | 20(4.5%) |
| Thr (T) number (ratio) | 25(5.7%) | 16(3.6%) |
| Tyr (Y) number (ratio) | 27(6.1%) | 24(5.4%) |
| Trp (W) number (ratio) | 9(2.0%) | 14(3.1%) |
| Val (V) number (ratio) | 22(5.0) | 23(5.2%) |
| Total number(ratio) of negatively charged residues^b^ | 57(12.9%) | 73(16.4%) |
| Total number(ratio) of positively charged residues^c^ | 43(9.7%) | 44(9.9%) |
| Total number(ratio) of polar neutral amino acids^d^ | 144(39.6%) | 127(28.5%) |

a Homology model of pBGL was generated using the SWISS-MODEL server with the β-glucosidase from Exiguobacterium antarcticum (EaBglA) as the structural template.

b Negatively charged residues include Asp (D) and Glu (E).

c Positively charged residues include Arg (R) and Lys (K).

d Polar uncharged amino acids include Ser (S), Thr (T), Asn (N), Gln (Q), Tyr (Y), and Cys (C).

**Figure S5. Overlap three-dimensional structural diagram of the psychrophilic enzyme (PDB ID: 5DT5) and pBGL.** Light blue represents pBGL and pink represents BGL.

**Figure S6. Conformational dynamics of psychrophilic enzyme 5DT5 (PDB ID: 5DT5) under different temperature conditions.** (A) RMSD values of 5DT5 at 300 K. (B) RMSD values of 5DT5 at 310 K. (C) RMSF profiles of pBGL WT, S306P, mBGL, and 5DT5 at 300 K. (D) RMSF profiles of the same four enzymes at 310 K. RMSF values were calculated from equilibrated molecular dynamics trajectories to evaluate residue-level conformational flexibility. Compared with mBGL and 5DT5, pBGL WT exhibited the largest increase in fluctuation upon temperature elevation, indicating greater temperature sensitivity of the cold-adapted enzyme. In contrast, mBGL showed relatively pronounced fluctuations mainly in loop regions, whereas most other structural regions displayed comparatively limited changes with increasing temperature.

**Figure S7. Statistical analysis of RMSD values for pBGL (WT) and mBGL at 300 K based on three independent MD simulations.** RMSD values during the equilibrium phase were averaged for each replica, and data are presented as mean ± SD. Statistical significance was evaluated using *t*-test. P < 0.001.

**Figure S8.** **(A-B) Porcupine plots illustrating the dominant collective motion described by the first eigenvectors of pBGL and mBGL at 300 K.** **(C-D) RMSF mapped onto the 3D backbones of pBGL and mBGL**. (A-B) Major motion modes of pBGL and mBGL, respectively. The two monomers are shown in cartoon representation. Arrows indicate the location of the active site. The cone length represents the magnitude of displacement derived from linear interpolation between the two extreme projections along the eigenvectors, highlighting structural differences between states rather than representing an actual transition pathway. (C-D) RMSF mapped onto the 3D backbones of pBGL and mBGL, respectively, at 300 K. Line thickness and color (red to blue) represent increasing fluctuation, with thin red lines corresponding to low RMSF and thick blue lines corresponding to high RMSF. Structures were visualized using UCSF Chimera.

**Figure S9. Structural comparison between the AlphaFold-predicted pBGL model and the SWISS-MODEL structure.** (A) Three-dimensional structural superposition of the AlphaFold-predicted model and the SWISS-MODEL structure. (B) Per-residue C_α_ distance between the two superimposed models. The two models showed high structural consistency, with a C_α_ RMSD of 1.866 Å over 429 aligned residues and 100% structural coverage.
